# Editorial: Mendelian randomization: an approach for precision medicine and public health

**DOI:** 10.3389/fgene.2025.1602592

**Published:** 2025-04-11

**Authors:** Xiaoqing Pan

**Affiliations:** Department of Mathematics, Shanghai Normal University, Shanghai, China

**Keywords:** mendelian randomization, genetic variants, causal inference, lifestyle factors, immune dysfunction

Mendelian randomization (MR) is an approach that leverages genetic variants as instrumental variables to infer causal relationships between exposures and health outcomes. By minimizing confounding and reverse causality, MR offers advantages over traditional observational studies to guide clinical decision-making and public health interventions. This Research Topic in *Frontiers in Genetics* showcases the versatility of MR, featuring studies that explore lifestyle factors, immune dysregulation in disease pathogenesis, and beyond.

Lifestyle factors significantly influence disease risk, yet observational studies often struggle to disentangle causation from correlation. This Research Topic includes several investigations that clarify these relationships using MR. Zhang et al. reported the association between coffee consumption and osteoarthritis risk, with body mass index (BMI) playing a significant mediating role (Zhang et al.). Their findings suggest that maintaining healthy BMI levels and choosing caffeinated over decaffeinated coffee varieties may help reduce osteoarthritis risk. Besides, Wang et al. explored the causal effects of smoking and alcohol consumption on upper urinary calculi, Liang et al. investigated tea intake and gout risk, and Zhou et al. identified positive causal effects of hypertension, BMI, waist-hip ratio adjusted for BMI and tobacco use to aortic aneurysm (Wang et al.; Liang et al.; Zhou et al.). All these studies exemplify how MR can validate or refute observational associations between lifestyle factors and diseases, offering insights into whether lifestyle habits directly contribute to joint health and reinforcing the importance of targeted public health campaigns to reduce disease risks.

The immune system’s role in disease pathogenesis is a recurring theme in this Research Topic. Utilizing MR approaches, Shi et al. discovered causal links between immune cell phenotypes (e.g., CD45 on HLA DR + NK cells) and cerebrovascular anomalies (Shi et al.). Inflammatory bowel diseases include ulcerative colitis and Crohn’s disease. Several studies investigated the causal links between inflammatory bowel disease and other diseases. Specifically, Xiao et al. investigated the causal links between inflammatory bowel disease and IgA nephropathy, offering mechanistic insights for comorbid conditions. However, no significant causal link was observed between inflammatory bowel disease and type 2 diabetes mellitus in Xiao et al., Tang et al.

MR’s applications extend far beyond lifestyle factors and immune dysregulation in disease pathogenesis, as demonstrated by the diverse range of studies in this Research Topic. For example, aging was a recurring theme in this Research Topic. Chen et al. reported earlier age at menarche may have a causal effect on the high risk of ischemic heart disease (Chen et al.). Xie et al. revealed that obstructive sleep apnea may accelerate telomere shortening, suggesting a biological pathway connecting sleep disorders to cellular aging (Xie et al.). Besides, the causality of biomarkers to disease outcomes remains an active area of investigation. Zhu et al. reported a causal relation rather than reversed causality between both hepatic enzymes Aspartate Aminotransferase (AST) and Alanine Aminotransferase (ALT) and spontaneous abortion, highlighting potential biomarkers to pregnancy complications (Zhu et al.). Guan et al. investigated the causal association between serum bilirubin and heart failure, yet not reaching statistical significance (Guan et al.). Zou et al. identified causal genes related with endoplasmic reticulum stress in inflammatory bowel disease (i.e., ulcerative colitis and Crohn’s disease) suggesting new therapeutic targets for clinical practice (Zou et al.). In addition, emerging evidence highlights important disease comorbidities across multiple conditions. Liu et al. reported an increased risk of heart failure in individuals with ankylosing spondylitis (Liu et al.). However, neither the causal relationship between ankylosing spondylitis and other cardiovascular diseases (such as atrial fibrillation, coronary artery disease), nor the reverse causality between ankylosing spondylitis and mentioned cardiovascular diseases, reached statistical significance. Shen et al. reported a bidirectional causal relationship between gastroesophageal reflux disease and chronic obstructive pulmonary disease (COPD), and COPD was also found to increase the risk of irritable bowel syndrome and constipation (Shen et al.). Shu et al. demonstrated a putative causal link of insomnia on low back pain and a null causal effect of low back pain on insomnia (Shu et al.). All these findings may stimulate new strategies for patient management in clinical practice, benefiting public health.

Collectively, this Research Topic highlights MR’s pivotal role in advancing precision medicine and public health. Continuous innovation in MR methodologies and interdisciplinary collaborations are encouraged to translate these insights into meaningful improvements for global health.

